# A Scoping Review of Current Social Emergency Medicine Research

**DOI:** 10.5811/westjem.2021.4.51518

**Published:** 2021-10-27

**Authors:** Ruhee Shah, Alessandra Della Porta, Sherman Leung, Margaret Samuels-Kalow, Elizabeth M. Schoenfeld, Lynne D. Richardson, Michelle P. Lin

**Affiliations:** * Icahn School of Medicine at Mount Sinai, New York, New York; † University of Miami Miller School of Medicine, Miami, Florida; ‡ Massachusetts General Hospital/Harvard Medical School, Department of Emergency Medicine, Boston, Massachusetts; § University of Massachusetts Medical School-Baystate, Department of Emergency Medicine, Springfield, Massachusetts; ¶ Icahn School of Medicine at Mount Sinai, Department of Emergency Medicine, New York, New York; || Icahn School of Medicine at Mount Sinai, Department of Population Health Science and Policy, New York, New York; # Icahn School of Medicine at Mount Sinai, Institute for Health Equity Research, New York, New York

## Abstract

**Introduction:**

Social emergency medicine (EM) is an emerging field that examines the intersection of emergency care and social factors that influence health outcomes. We conducted a scoping review to explore the breadth and content of existing research pertaining to social EM to identify potential areas where future social EM research efforts should be directed.

**Methods:**

We conducted a comprehensive PubMed search using Medical Subject Heading terms and phrases pertaining to social EM topic areas (e.g., “homelessness,” “housing instability”) based on previously published expert consensus. For searches that yielded fewer than 100 total publications, we used the PubMed “similar publications” tool to expand the search and ensure no relevant publications were missed. Studies were independently abstracted by two investigators and classified as relevant if they were conducted in US or Canadian emergency departments (ED). We classified relevant publications by study design type (observational or interventional research, systematic review, or commentary), publication site, and year. Discrepancies in relevant publications or classification were reviewed by a third investigator.

**Results:**

Our search strategy yielded 1,571 publications, of which 590 (38%) were relevant to social EM; among relevant publications, 58 (10%) were interventional studies, 410 (69%) were observational studies, 26 (4%) were systematic reviews, and 96 (16%) were commentaries. The majority (68%) of studies were published between 2010–2020. Firearm research and lesbian, gay, bisexual, transgender, and queer (LGBTQ) health research in particular grew rapidly over the last five years. The human trafficking topic area had the highest percentage (21%) of interventional studies. A significant portion of publications -- as high as 42% in the firearm violence topic area – included observational data or interventions related to children or the pediatric ED. Areas with more search results often included many publications describing disparities known to predispose ED patients to adverse outcomes (e.g., socioeconomic or racial disparities), or the influence of social determinants on ED utilization.

**Conclusion:**

Social emergency medicine research has been growing over the past 10 years, although areas such as firearm violence and LGBTQ health have had more research activity than other topics. The field would benefit from a consensus-driven research agenda.

## INTRODUCTION

### Background and Importance

In 1848 Rudolph Virchow declared social problems to be “largely within the jurisdiction” of physicians.^1^,^2^ Emergency physicians serve as safety net providers and are often on the front line of epidemics, natural disasters, and civil unrest.^3^ The emergency department (ED) is a unique place to identify and intervene in social issues, as patients often present with complaints directly influenced by social determinants of health (SDOH),^4^ and EDs serve patients who have limited access to care.^5^ As a result, the field of social emergency medicine (EM) has developed to examine and influence social factors in the context of acute healthcare needs. The scope of social EM is immense, including domains from housing insecurity to substance use, to gun and intimate partner violence, and many others. Many domains within social EM are known to influence emergency care utilization and health outcomes.

### Goals of This Investigation

While prior systematic reviews have examined the existing literature with a specific focus on material needs, there is a need to characterize the literature examining the broader field of social factors, including non-material factors – such as language, exposure to violence, and immigration status – known to influence emergency care and outcomes.^6^ The primary aims of this scoping review were to understand and map the breadth of current literature for various social EM topics and categorize the type of research that exists for each topic, in order to identify potential areas where future social EM research efforts should be directed.

## METHODS

This review was informed by the Preferred Reporting Items for Systematic Review and Meta-analysis (PRISMA) guidelines for scoping reviews. We identified 11 content areas based on a previously published systematic review of patients’ social and economic needs, including housing needs, employment needs, education and literacy, financial insecurity, personal safety (including intimate partner violence, human trafficking, firearms, child abuse, and elder abuse), and food insecurity.^7^ Additional topic areas were added based on author consensus, including lesbian, gay, bisexual, transgender, and queer (LGBTQ) health, language, immigration, incarceration, and transportation needs. Two final search terms (“social determinants of health” and “social emergency medicine training”), were added in consultation with a research librarian to ensure inclusion of publications that address more than one topic, as well as educational research.

We conducted a comprehensive literature search using a combination of Medical Subject Heading (MeSH) terms and phrases pertaining to topic areas (eg, “homelessness,” “housing instability”). We restricted studies to those conducted in the US or Canada. Given the focus on social EM, we included the MeSH terms (((“Emergency Service, Hospital”[Majr]) OR (emergency (room[Title] OR department[Title] OR medicine[Title] OR care[Title] OR visit[Title])))). A full list of search terms can be found in Appendix A.

We used the PubMed database for our searches, with the exception of the “Social Emergency Medicine Training” search, which also used the MedEd Portal database. For searches that yielded fewer than 100 total publications, we used the PubMed “similar publications” tool to expand the search and ensure no relevant publications were missed. Criteria for inclusion were as follows: (1) published in English; (2) conducted in the US or Canada through July 31, 2020; and (3) deemed relevant to social EM. Studies were considered relevant to social EM if they focused on social factors in the context of acute healthcare needs; therefore, we included the following criteria: 1) the study population consisted of ED patients or emergency clinicians; 2) the study or intervention occurred in the ED; or (3) ED utilization or outcomes were defined as a primary outcome.

Once a publication was deemed to meet inclusion criteria we extracted additional information such as title, PubMed ID, year of publication, and study design type (original observational or interventional research, systematic review, or commentary) into a standardized data collection form. We further catalogued observational and interventional publications by setting (single center, multicenter regional, and multicenter national). For each publication, study objectives (eg, defining prevalence, evaluating an educational intervention) were also recorded. For search results in each topic area, two co-investigators independently assessed each study for inclusion and relevance to social EM, Any discrepancies in relevance or categorization were reviewed and reconciled by a third reviewer. We also classified publications classified as relating to pediatric populations if they included children or adolescents (≤ 21 years) or if they were conducted in pediatric EDs.

## RESULTS

**Figure 1:**
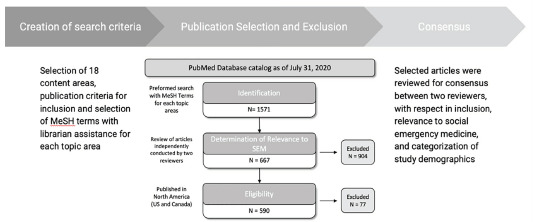
Publication selection and exclusion for all topic areas.

**Figure 2:**
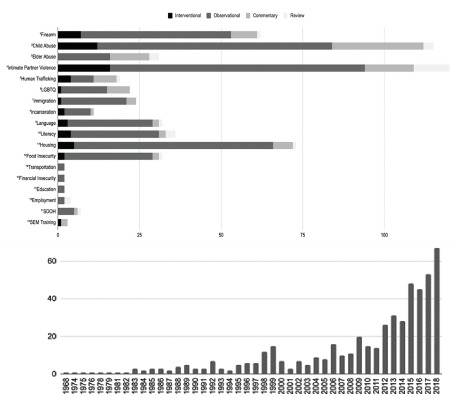
Summary of results across all categories by article type and number of publications by year. Top: 1. Observational: 74%, Interventional: 11%, Systematic Review: 2%, Commentary: 13% 2. Observational: 63%, Interventional: 10%, Systematic Review: 10%, Commentary: 24% 3. Observational: 52%, Interventional: 0%, Systematic Review: 10%, Commentary: 39% 4. Observational: 65%, Interventional: 13%, Systematic Review: 9%, Commentary: 13% 5. Observational: 42%, Interventional: 15%, Systematic Review: 5%, Commentary: 37% 6. Observational: 64%, Interventional: 5%, Systematic Review: 0%, Commentary: 32% 7. Observational: 83%, Interventional: 4%, Systematic Review: 0%, Commentary: 13% 8. Observational: 73%, Interventional: 18%, Systematic Review: 0%, Commentary: 9% 9. Observational: 81%, Interventional: 9%, Systematic Review: 3%, Commentary: 6% 10. Observational: 75%, Interventional: 11%, Systematic Review: 8%, Commentary: 6% 11. Observational: 84%, Interventional: 7%, Systematic Review: 1%, Commentary: 8%12. Observational: 87%, Interventional: 6%, Systematic Review: 3%, Commentary: 6% 13. Observational: 100%, Interventional: 0%, Systematic Review: 0%, Commentary: 0% 14. Observational: 100%, Interventional: 0%, Systematic Review: 0%, Commentary, 0%, 15. Observational: 100%, Interventional, 0%, Systematic Review, 0%, Commentary, 0% 16. Observational: 50%, Interventional: 0%, Systematic Review: 50%, Commentary: 0% 17. Observational: 63%, Interventional: 0%, Systematic Review: 13%, Commentary: 25% 18. Observational: 0%, Interventional: 33%, Systematic Review: 0%, Commentary: 67%

Our search strategy identified 1571 publications, of which 590 publications in 18 categories were classified as relevant to social EM. Depiction of search strategy and classification process are in Figure 1. The study designs of included publications were as follows: 58 (10%) interventional publications; 410 (69%) observational publications; 26 (4%) systematic reviews; and 96 (16%) commentaries. Publication years ranged from 1968 to 2020, with 402 (68%) eligible articles published since 2010. Results are summarized in Figure 2. Study objectives within each topic are summarized in the Table.

**Figure 3:**
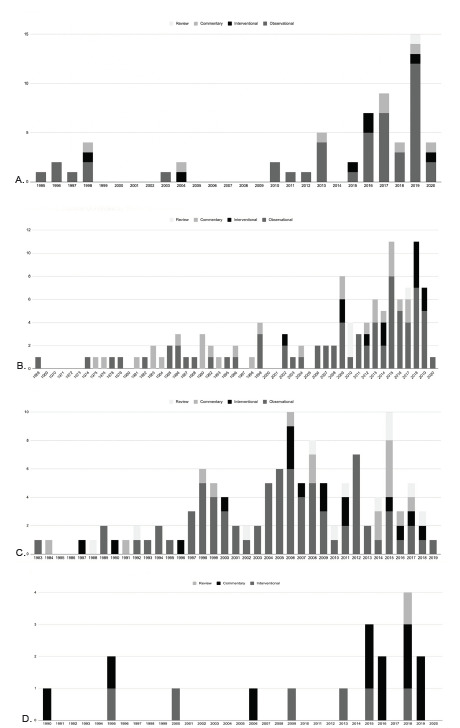
A–D. Depiction of publication type and timeline of publications for A. firearm, B. child abuse, C. interpersonal violence, and D. homelessness topic areas in the social emergency medicine literature.

Figures 3A through 3D show study type by year for select topics with the largest number of studies (firearms, intimate partner violence, child abuse, and housing/homelessness).

### Firearms

We identified 62 relevant publications 8–69: 46 observational studies; seven interventional studies^18^,^38^,^55^,^60^,^64^,^66^,^67^; one systematic review^15^; and eight commentaries (Figure 3A).^22^,^23^,^33^,^46^,^56^,^62^,^65^,^69^ Two-thirds of these publications were published between 2015–2020. Of the observational studies, nine (20%) publications focused on psychiatric issues; specifically, they focused on lethal means counseling and access to firearms among patients presenting with suicidal ideation.^10^,^11^,^25^,^26^,^37^,^38^,^45^,^50^,^63^ Twenty-four publications attempted to characterize firearm violence, studying the prevalence of firearm access (2%)^51^ and injuries (15%),^24^,^27^,^41^,^44^,^48^,^57^,^68^ behavioral risk factors for firearm violence (11%),^9^,^12^,^13^,^31^,^42^ characteristics of patients presenting for firearm injuries (24%),^14^,^17^,^21^,^30^,^36^,^39^,^40^,^52^–^54^,^58^ and the severity of firearm injuries (4%).^34^,^35^ Two studies (4%) looked into developing screening tools to predict future risk of firearm violence,^22^,^31^ and five (11%) assessed patient and provider attitudes toward asking about firearm access and safety in the ED.^19^,^20^,^45^,^47^,^50^ Forty-two percent of publications focused on pediatric ED patients. A plurality of interventional studies (43%) focused on lethal means counseling.^38^,^55^,^66^

### Child Abuse

We identified 114 relevant publications: 71 observational studies^70^–^141^; 12 interventional studies^142^–^153^; three review publications^154^–^156^; and 28 commentary publications (Figure 3B).^157^–^184^ There were several common objectives among the observational studies. Twenty-two (31%) observational publications focused on determining incidence/prevalence of child abuse in different settings (single EDs, specific geographic areas, nationwide), and characterizing cases of child abuse.^70^,^76^,^82^,^83^,^88^,^93^,^95^,^97^,^98^,^102^,^113^,^116^,^119^,^120^,^124^,^125^,^127^,^129^,^131^,^132^,^136^,^138^ Child abuse cases were often categorized by demographic characteristics, such as age, gender, race, and insurance status, as well as injury patterns. Nineteen (26%) studies focused specifically on injury patterns of abused children, and the likelihood of child abuse among patients presenting with fractures, head trauma, and oral injuries.^71^,^75^,^81^,^82^,^86^,^91^,^95^,^97^,^99^,^101^,^107^,^109^–^111^,^113^,^115^,^117^,^119^,^127^ About 22 (31%) studies focused specifically on child sexual assault cases,^70^,^79^,^80^,^96^,^98^,^106^,^112^,^116^,^118^,^120^,^123^,^125^,^130^,^132^,^133^,^141^ with six of these studies looking at sexually transmitted infection (STI) and pregnancy testing, STI prophylaxis, and the use of sexual assault nurse examiners.^77^,^78^,^121^,^122^,^140^,^150^ Two of three review publications focused on screening,^154^,^155^ with one publication focusing on improving the ED workflow for suspected or confirmed child abuse cases.^156^

Other common study objectives included examining and amending the ED workflow for child abuse cases, developing screening protocols, and understanding provider knowledge and training with regard to child abuse in the ED. A plurality (42%) of the interventional studies involved evaluations of educational interventions for ED providers meant to improve child abuse screening and recognition.^142^,^144^,^147^,^149^,^152^ Three (25%) interventional studies focused on child sexual assault.^142^,^144^,^150^

### Elder Abuse

We identified 31 relevant publications: 16 observational studies^185^–^215^; three review publications^187^,^212^,^214^; and 12 commentary publications.^185^,^186^,^192^,^195^,^197^,^198^,^200^,^201^,^205^,^210^,^211^,^215^ Common objectives among the observational studies included the following: developing and testing screening tools (N = 5, 31%)^194^,^196^,^202^,^204^,^206^; ED utilization by abused patients (N = 2, 13%)^190^,^207^; injury patterns among abused patients (13%)^191^,^208^,^213^; patient characteristics (N = 2, 13%)^202^,^209^; provider awareness and perspectives on elder abuse (N = 2, 13%)^188^,^203^; and prevalence of elder abuse (N = 1, 6%).^189^ There was a lack of interventional studies regarding educational interventions or the use of screening tools. All 12 commentary publications from the 1990s to 2019 served to raise awareness about elder abuse in the ED and ways to identify and combat it.

### Intimate Partner Violence

We identified 120 relevant publications: 78 observational studies^216^–^293^; 16 interventional studies^294^–^309^; 11 review publications^310^–^320^ ; and 15 commentary publications (Figure 3C).^321^–^335^ The most prevalent objectives among original research studies were intimate partner violence (IPV) screening (N = 20, 26%)^218^,^226^,^229^,^230^,^237^,^240^,^242^,^245^,^246^,^258^,^259^,^261^,^264^,^270^,^273^,^278^,^281^,^293^,^300^,^306^; characteristics and risk factors (N = 15, 19%)^220^,^221^,^223^,^227^,^228^,^231^,^233^,^242^,^247^,^249^,^262^,^263^,^279^,^284^,^292^; substance use and mental health associations (N = 14, 18%)^216^,^217^,^224^,^225^,^228^,^238^,^239^,^241^,^250^,^255^,^268^,^282^,^283^,^302^; prevalence of IPV (N = 12, 15%)^219^,^221^,^222^,^236^,^242^,^245^,^257^,^263^,^271^,^276^,^284^,^286^; provider perspectives on IPV screening and protocols (N = 8, 10%)^232^,^235^,^251^,^252^,^254^,^256^,^265^,^267^; and patient perspectives on the acceptability of IPV screening and discussion in the ED (N=9, 12%).^242^,^243^,^252^,^265^,^267^,^269^,^272^,^277^,^289^ Five studies focused specifically on IPV screening for caregivers of pediatric patients (6%).^242^,^248^,^266^,^298^,^303^ There were also three studies focused on perpetrators of IPV.^234^,^253^,^290^

Of the 16 interventional studies, nine (56%) were related to screening,^294^,^296^–^298^,^300^,^303^,^304^,^306^,^308^ three (19%) were related to addressing substance use among patients with co-existing IPV,^299^,^302^,^307^ and two (13%) were educational interventions for ED staff.^303^,^309^

### Human Trafficking

We identified 19 relevant publications: four interventional studies^336^–^339^; seven observational studies^79^,^340^–^345^; one systematic review^346^; and seven commentary publications.^347^–^353^ All publications were published after 2012. Of the seven observational studies, three (43%) related to screening tools to identify patients experiencing sex trafficking.^340^,^342^,^344^ Two (25%) focused on patient characteristics,^79^,^345^ one was a case report (13%),^343^ and the other study focused on emergency nurses’ perspectives (13%).^341^ All four interventional studies looked at the efficacy of educational modules on ED staff in better understanding the issue of human trafficking in the ED and being better able to identify human trafficking victims in the ED. The systematic review was of existing human trafficking screening tools in the ED. Seven studies (37%) focused specifically on child sex trafficking victims in the ED.^340^,^344^–^346^,^352^,^353^

### Lesbian, Gay, Bisexual, Transgender, and Queer Health

We identified 22 relevant publications: 14 observational studies^354^–^367^; one interventional study^368^; and seven^369^–^375^ commentary publications. Of these, 21 (95%) were published after 2014. Of the observational studies, five (36%) focused on patient provider attitudes toward sexual orientation and gender identity data collection in the ED,^354^,^355^,^361^,^362^,^365^ and six (43%) focused on the care of transgender patients in the ED,^356^,^358^–^360^,^367^,^370^ with many surveying experiences of discrimination among transgender patients.^358^,^360^,^363^,^367^ Four (29%) observational publications focused on LGBTQ health competency training by emergency care providers.^356^,^364^,^366^,^368^,^373^ One (7%) publication broke down intimate partner violence prevalence in the ED by the sexual orientation of patients.^357^ The commentary publications centered on the same themes.

The single interventional publication used pre/post data to evaluate the efficacy of an ED competency training in LGBTQ health.^368^

### Immigration

We identified 24 relevant publications:^376^–^399^ 20 observational studies^376^–^395^; one interventional study^399^; and three commentary publications.^396^–^398^ All observational publications investigated ED utilization in immigrant vs non-immigrant groups, with some specifically assessing Latino populations. Two publications (10%) studied the fear of ED utilization among Latino populations.^376^,^386^ The single interventional study assessed a texting-based intervention of Latino families as a means to reduce ED utilization while increasing well-care and vaccine adherence.^399^

### Incarceration

We identified 11 relevant publications: eight observational studies^400^–^407^; two interventional studies^408^,^409^; and one commentary publication.^410^ Of the observational studies, five (63%) publications centered on ED utilization after release from prison.^400^,^403^–^405^,^407^ Both interventional publications focused on models of care for recently released prisoners. Of all publications, three (38%) focused on pediatric populations.^401^,^402^,^404^

### Language

We identified 32 relevant publications 411–442; 26 observational studies^411^–^436^; three interventional studies^437^–^439^; one review publication; and two commentary publications. The observational research spanned a broad range of topic areas covering many parts of ED care, including triage (8%),^419^,^438^ history of present illness collection (4%),^416^ management of care (4%),^411^ interpreter utilization and need (12%),^415^,^428^,^429^ ED resource utilization (15%),^423^,^424^,^431^,^433^ length of stay (8%),^427^,^430^ the discharge process (15%),^417^,^420^–^422^ and follow-up care (8%).^432^,^435^ Of the interventional studies, one examined the role of the patient’s preferred language in the success of a drinking intervention.^437^ Another looked at the efficacy and efficiency of a bilingual, kiosk-based self-triage system compared to a nurse.^438^ The third publication investigated the effectiveness of a bilingual medical history questionnaire.^439^ The review and commentary pieces described the language barriers patients face in the ED^440^ and utilization of interpreter services.^441^,^442^ Of all publications, 12 (38%) focused on pediatric populations.^411^,^417^,^420^–^422^,^425^–^427^,^430^,^431^,^433^,^438^

### Literacy

We identified 34 relevant publications^443^–^475^: 25 observational studies^443^–^466^; four interventional studies^467^–^470^; three review publications^473^–^475^; and two commentary publications.^471^,^472^ Of the observational studies, 11 (41%) examined health literacy screening and patients’ understanding of discharge instructions,^443^,^445^,^451^–^453^,^461^–^463^,^466^,^474^,^476^ eight (30%) investigated the relationship between health literacy and ED utilization,^447^,^448^,^455^,^457^,^458^,^460^,^469^,^473^ and 10 (37%) focused on the literacy of the parents of pediatric patients.^446^,^448^,^454^,^457^,^458^,^467^–^469^,^473^,^475^ One study focused on ways to improve a patient’s understanding of the clinical encounter with improved communication tools for physicians or teach-back strategies with patients.^450^ All four of the interventional studies involved educational interventions for parents of pediatric patients.^467^–^470^

### Housing/Homelessness

We identified 73 relevant publications^4^,^477^–^548^: 61 observational studies^4^,^477^–^536^; five interventional studies^537^–^541^; six commentary publications,^542^–^547^; and one review publication (Figure 3D).^548^ Twenty-eight (46%) observational studies focused on ED utilization, including factors predicting ED utilization and characteristics of homeless patients that frequently used the ED.^4^,^478^,^479^,^487^,^492^,^499^,^501^–^503^,^505^–^508^,^510^,^511^,^513^,^514^,^519^,^521^,^524^,^527^–^533^ Another common study objective (16%) included the effect of substance use and mental illness on ED utilization of homeless patients.^480^,^484^,^488^,^493^,^494^,^500^,^509^,^526^,^535^,^539^ Four (7%) observational studies looked into ED provider perspectives,^515^,^520^,^523^ and two looked into homeless patient perspectives on ED services.^482^,^483^ A few studies focused on specific sub-populations of homeless patients, including veterans,^492^,^519^,^524^,^528^ older adults,^499^,^501^ and pediatric patients.^485^,^486^,^507^,^534^ Two (40%) interventional studies centered on analyzing the effect of case management interventions on ED utilization.^537^,^541^

### Food Insecurity

We identified 29 relevant publications 25 observational studies^549^–^574^; two interventional studies,^575^,^576^; and two commentary publications.^577^,^578^ Objectives among observational studies included the following: food insecurity prevalence (27%)^555^,^560^–^562^,^564^,^565^,^567^; ED utilization (19%)^549^,^550^,^552^,^570^,^571^; screening (8%),^553^,^572^; and cost of care (12%).^550^,^559^,^561^ Four (15%) publications explored the health consequences of food insecurity.^561^,^565^–^567^ Five (19%) publications focused the intersection between diabetic patients, food insecurity, and presentation to the ED.^552^,^554^,^559^,^563^,^568^ Three publications (12%) also focused specifically on Supplemental Nutrition Assistance Program benefits running out near the end of the month,^569^ and the impact on patients with diabetes^568^ and hypertension.^556^ Nine (35%) observational studies focused on pediatric populations.^549^,^553^–^555^,^560^,^562^,^566^,^573^,^574^ One interventional study was a randomized controlled trial of two screening methods,^576^ and the other was a program to improve access to food for pediatric ED patients.^575^

### Transportation

Two relevant publications were identified,^579^,^580^ both of which were observational and published in 2019. One publication compared proximity of freestanding EDs and hospital EDs to public transit in three different metro areas.^579^ The second discussed ridesharing services as alternative options to ambulances for stable psychiatric patients to reach the emergency department.^580^

### Financial Insecurity

We identified two relevant publications, both of which were observational.^581^,^582^ Both publications focused on the financial burden for patients of specific chief complaints in the ED, including atopic dermatitis and orthopedic injuries. One publication looked specifically at the pediatric population.^582^

### Education

Two relevant publications were identified, both of which were observational studies. One publication explored the association between educational attainment and patterns of ED use in patients with sickle cell disease,^583^ and the other focused on the relationship between educational attainment and likelihood of receiving opioids for pain management in the ED.^584^

### Employment

We identified three relevant publications: two observational studies^585^,^586^; and one systematic review.^587^ The systematic review broadly examined social and demographic characteristics influencing ED use, and included unemployment as one of many variables. Of the observational studies, one correlated unemployment rates and trauma admissions in New Orleans,^586^ and the other correlated ED visits with areas experiencing “economic hazard,” which included unemployment rate.^585^

### Social Determinants of Health (SDOH)

We identified seven relevant publications^3^,^6^,^555^,^588^–^593^: five observational studies^3^,^588^–^590^,^593^; one review publication^6^; and one commentary publication.^592^ There were no interventional studies. Three (60%) of the observational publications focused on the SDOH of specific populations – dialysis patients,^588^ patients with sickle cell disease,^589^ and patients who inject intravenous drugs^593^ – and the relationship with ED utilization. Another publication focused on predicting ED visits using SDOH measures.^590^ Two publications (29%) focused on pediatric populations.^555^,^590^

### Social Emergency Medicine Training

A total of three relevant publications were identified: one educational intervention^594^; and two commentary publications.^591^,^595^ The education intervention assessed the impact of a longitudinal curriculum for fourth-year medical students on their EM clerkship rotation.^594^ The commentary publications discussed the incorporation of SDOH into various aspects of EM training.

## DISCUSSION

We identified 590 publications in 18 categories relevant to social EM, demonstrating a high degree of interest in social EM topics. Despite the large and growing number of relevant publications across categories, only 58 publications (10%) were interventional studies. In most topic areas, observational studies have already done a thorough job of describing and characterizing disparities by social identity and circumstance. For example, while a large number of studies looked at ways to effectively screen patients for things like interpersonal violence, health literacy, and human trafficking, there were few publications following up on outcomes for patients who screened positive. Even fewer interventional studies examined patient-oriented outcomes; most interventional studies were educational in nature, with outcomes such as clinician awareness and effectiveness of screening. The dearth of interventional studies examining patient outcomes underscores a need for funding to support testing and dissemination of potential interventions, given that observational studies are more feasible and less resource-intensive than interventional studies.

Topics with the most published research included gun violence, child abuse, intimate partner violence, and housing/homelessness; these four categories combined constituted 63% of all relevant publications. There were several topic areas in which the literature base has grown rapidly in recent years, including gun violence and LGBTQ health. Topics such as elder abuse and incarceration have been the topic of few publications in the last five years, suggesting possible stagnation in these areas. About one third of the relevant publications included were related to the pediatric ED. We found very little research in the following eight topic areas: transportation, financial insecurity, education, employment, incarceration, racism, and legal needs, possibly because they may have been traditionally perceived as less directly related to clinical care and may thus have received less attention.

Prior literature has examined the scope of EM research focused on material needs; our study also examines non-material social risk factors for health outcomes. While the acknowledgment of the interplay between social factors and patients’ acute health care needs and outcomes has existed in medical literature for decades, terminology such as “social emergency medicine” is more recent and has increased following a consensus conference about the field.^7^

## LIMITATIONS

There were several limitations to our review. First, we largely used only the PubMed database, which may have left out relevant publications; however, we systematically searched PubMed, and a majority of biomedical publications are indexed in PubMed. All our search terms were specific to EM, which may have also left out research relevant to EM conducted in related settings or fields. We limited our search to “title only” rather than “title and abstract,” which may have also omitted relevant publications; however, after attempting both “title only” and “title and abstract” searches, we found “title only” searches to have much higher relevance. We also did not conduct a detailed analysis of publication quality, given that we set out to complete a scoping review rather than a systematic review; however, publication quality would have been difficult to assess across the diversity of topic areas given the vast array of topics and study designs. We maximized reliability by using two independent reviewers for each topic area, with a third reviewer who reconciled any differences in opinion regarding relevance or publication inclusion.

## CONCLUSION

Social emergency medicine research has accelerated in recent years. Numerous observational studies and commentary publications have defined and characterized problems relevant to social EM, and several educational interventions have demonstrated ways to improve provider awareness of different social EM topics. However, based on our review, there is a dearth of social EM research focused on patient-centered interventions. A consensus-driven research agenda should be pursued to accelerate patient-centered interventions aimed at social factors that influence acute healthcare and outcomes.

## Supplementary Information



## Figures and Tables

**Table:** Number of included publications and their most frequent study objectives in the social emergency medicine literature.

Topic area (590)	Study objectives
Firearms (62)	Prevalence Patient characteristics Risk factors for violence Severity Screening Psychiatric (Lethal means counseling) Patient and provider perspectives towards discussing firearm safety
Child abuse (114)	Prevalence Patient characteristics Injury patterns Sexual assault Screening Provider knowledge/training Educational interventions
Elder abuse (31)	Prevalence Patient characteristics Screening ED utilization Injury patterns Provider knowledge
Intimate partner violence (120)	Prevalence Screening Patient characteristics Risk factors Psychiatric (substance use/mental health) Patient and provider perspectives on IPV screening Educational interventions
Human trafficking (19)	Patient characteristics Screening Educational interventions
Lesbian, gay, bisexual, transgender, and queer health (22)	Prevalence of IPV Care of transgender patients Patient and provider attitudes towards sexual orientation and gender identity data collection Competency training Educational intervention
Immigration (24)	ED utilization Preventative care intervention
Incarceration (11)	ED utilization (post-release) Models of Care (interventional)
Language (32)	Aspects of ED care (triage, HPI, management of care, interpreter utilization, ED resource utilization, length of stay, discharge, follow-up care) Effectiveness of bilingual triage/medical history (interventional)
Literacy (34)	Screening (literacy and health literacy) Understanding discharge instructions ED utilization Communication tools Educational interventions (parents of pediatric patients)
Housing/homelessness (73)	ED utilization Patient characteristics Psychiatric (substance use and mental health) Patient and provider perspectives Case management interventions
Food insecurity (29)	Prevalence ED utilization Screening Cost of care Health effects of food insecurity Diabetes SNAP and chronic illness Food access intervention
Transportation (2)	ED access Psychiatric patients
Financial insecurity (2)	Financial burden of specific chief complaints
Education (2)	ED utilization Pain management
Employment (3)	ED utilization
Social determinants of health (8)	ED utilization
SEM training (3)	Educational Intervention

*ED*, emergency department, *IPV*, intimate partner violence; *HPI*, history of present illness.

*ED*, emergency department; *SNAP*, Supplemental Nutrition Assistance Program; *SEM*, social emergency medicine.
