# Supplementary material for: A Scoping Review of Current Social Emergency Medicine Research
Source: West J Emerg Med. 2021 Oct 27;22(6):1360–8. doi: 10.5811/westjem.2021.4.51518 (PMC8597693; doi:10.5811/westjem.2021.4.51518)
Supplement: Supplementary file 1 [file wjem-22-1360-s001.docx]

**APPENDIX**

We conducted a comprehensive PubMed search using a combination of MeSH terms and phrases pertaining to topic areas (Ex. “homelessness,” “housing instability”). We restricted studies to those conducted in the US or Canada. Given the focus on social emergency medicine, we included the MeSH terms ((("Emergency Service, Hospital"[Majr]) OR (emergency (room[Title] OR department[Title] OR medicine[Title] OR care[Title] OR visit[Title])))) in each search, in addition to the topic specific terms. The full list of search terms for each topic area is listed in detail below.

| Topic | MeSH Search Criteria |
| --- | --- |
| Firearm | ((("Emergency Service, Hospital"[Majr]) OR (emergency (room[Title] OR department[Title] OR medicine[Title] OR care[Title] OR visit[Title])))) AND (((("Firearms"[Majr]) OR (gun[Title] OR guns[Title] OR firearm*[Title]))) NOT (nail or nail gun)) |
| Child Abuse | (((((("Emergency Service, Hospital"[Majr] OR (emergency (room[Title] OR department[Title] OR medicine[Title] OR care[Title] OR visit[Title])))))))) AND (((("child abuse"[Majr]))))) |
| Elder Abuse | (((((("Emergency Service, Hospital"[Majr] OR (emergency (room[Title] OR department[Title] OR medicine[Title] OR care[Title] OR visit[Title])))))))) AND (((("elder abuse"[Majr])))) |
| Intimate Partner Violence | (((((("Emergency Service, Hospital"[Majr] OR (emergency (room[title] OR department[Title] OR medicine[Title] OR care[Title] OR visit[Title])))))))) AND (((("intimate partner violence"[Majr]))))) |
| Human Trafficking | ((("Emergency Service, Hospital"[Majr]) OR (emergency (room[Title] OR department[Title] OR medicine[Title] OR care[Title] OR visit[Title])))) AND "Human Trafficking"[Majr] |
| LGBTQ Health | ((("Emergency Service, Hospital"[Majr]) OR (emergency (room[Title] OR department[Title] OR medicine[Title] OR care[Title] OR visit[Title])))) AND ("transgender"[Title] OR "gender orientation"[Title] OR "sexual orientation"[Title] OR "LGBTQ"[Title]) |
| Immigration | ((("Emergency Service, Hospital"[Majr]) OR (emergency (room[Title] OR department[Title] OR medicine[Title] OR care[Title] OR visit[Title])))) AND ("Emigrants and Immigrants"[Majr] OR "Undocumented Immigrants"[Majr] OR "deport*") |
| Incarceration | ((("Emergency Service, Hospital"[Majr]) OR (emergency (room[Title] OR department[Title] OR medicine[Title] OR care[Title] OR visit[Title])))) AND ("incarcerated"[Title] OR "prison"[Title] OR "corrections"[Title] OR "detention"[Title] OR "justice involved"[Title]) |
| Language | (((("Emergency Service, Hospital"[Majr])) OR (emergency (room[Title] OR medicine[Title] OR care[Title] OR visit)[Title]))) AND (language[Title] OR bilingual[Title] OR multilingual[Title] OR translator[Title] OR interpreter[Title] OR translation services[Title] OR interpretation services[Title]) NOT “natural language processing” |
| Literacy | ((("Emergency Service, Hospital"[Majr]) OR (emergency (room[Title] OR department[Title] OR medicine[Title] OR care[Title] OR visit[Title])))) AND ("Health Literacy"[Majr] OR "Literacy"[Majr] OR "Comprehension"[Majr]) |
| Homelessness/ Housing | ((("Emergency Service, Hospital"[Majr]) OR (emergency (room[Title] OR department[Title] OR medicine[Title] OR care[Title] OR visit[Title])))) AND ("housing instability" OR "Homeless Persons"[Majr]) |
| Food Insecurity | ((("Emergency Service, Hospital"[Majr]) OR (emergency (room[Title] OR department[Title] OR medicine[Title] OR care[Title] OR visit[Title])))) AND ("food insecurity"[Title/Abstract] OR "hunger"[Title/Abstract] OR "food security"[Title/Abstract]) |
| Transportation | (((("Emergency Service, Hospital"[Majr]) OR (emergency (room[Title] OR department[Title] OR medicine[Title] OR care[Title] OR visit[Title])))) AND ("public transportation"[Title/Abstract] OR "non-emergency transport*"[Title/Abstract] OR "accessible transport*"[Title/Abstract] OR subway[Title/Abstract])) NOT (accident[Title/Abstract] OR crash[Title/Abstract]) |
| Financial Insecurity | ((("Emergency Service, Hospital"[Majr]) OR (emergency (room[Title] OR department[Title] OR medicine[Title] OR care[Title] OR visit[Title])))) AND ("financial insecurity"[Title] OR "financial burden"[Title] OR "government assistance"[Title] OR "government benefits"[Title] OR "bankrupt*"[Title] OR "welfare program"[Title]) |
| Education | ((("Emergency Service, Hospital"[Majr]) OR (emergency (room[Title] OR department[Title] OR medicine[Title] OR care[Title] OR visit[Title])))) AND ("educational attainment"[Title] OR "education level"[Title] OR "education status"[Title]) |
| Employment | ((("Emergency Service, Hospital"[Majr]) OR (emergency (room[Title] OR department[Title] OR medicine[Title] OR care[Title] OR visit[Title])))) AND unemployment[Title] |
| Social Determinants of Health | ((("Emergency Service, Hospital"[Majr]) OR (emergency (room[Title] OR department[Title] OR medicine[Title] OR care[Title] OR visit)[Title]))) AND (("Social Determinants of Health"[Majr]) OR social determinants[Title]) |
| SEM Training | “Social Emergency Medicine Training” |
